# On the Therapeutics of Acute Rheumatism

**Published:** 1880-01

**Authors:** Roberts Bartholow

**Affiliations:** Professor of Therapeutics and Materia Medica in Jefferson Medical College, Philadelphia


[From the Medical News and Abstract.]

### ON THE THERAPEUTICS OF ACUTE RHEUMATISM.

A CLINICAL LECTURE DELIVERED AT THE JEFFERSON COLLEGE HOSPITAL.

BY ROBERTS BARTHOLOW, M. D.,
Professor of Therapeutics and Materia Medica in Jefferson Medical College, Philadelphia.

GENTLEMEN :—In no disease is the influence of fashion in therapeutics more conspicuous than in the treatment of acute rheumatism. Now it is a therapeutical nihilism, as the "mint-water method" at Guy's Hospital; again it is the application of blisters to the affected joints; now it is the alkaline treatment; again it is salicylic acid.

Whatever it may be, the remedy has almost universal sway for a time, until supplanted by some other fashion. I need hardly say that we should not abandon an old and well-tried remedy for a new one, simply because it is new; but the new one should be distinctly better. It may be useful, then, in view of the cases which have been before us, to examine into this subject of the therapeutics of acute rheumatism, and come to some conclusions, if we can, in regard to the relative merits of the various remedies which have occupied professional attention for several years past.

First of all, gentlemen, I cannot too strongly insist on this fundamental fact, that no single remedy can be rightly applied to every case of acute rheumatism. In this disease, notwithstanding it pursues a pretty uniform plan, there are wide differences in origin, in the type of individual cases, and in the constitutional state and bodily condition of patients—all of which must have due recognition if we would employ our therapeutical expedients wisely. Let me illustrate: Rheumatism seems sometimes to be of distinctly nervous origin. We now know that certain changes in the spinal cord, and injuries of nerves, are followed by joint inflammations similar to those of acute rheumatism. Again, the circulation of some organic acid in the blood has seemed to excite rheumatic inflammation; at least we know that the sweat and the urine are very acid, that endocarditis has been excited by injecting lactic acid into the peritoneal cavity of animals, and that rheumatic attacks have been induced by the administration of lactic acid for diabetes.

Furthermore, the most superficial inspection of the cases which have been shown must have satisfied you that there are three classes of subjects who are attacked by rheumatism: the cachectic, feeble, and nervous; the obese, florid but flabby, drinkers of malt liquors; the vigorous and able-bodied, who have inherited or acquired a rheumatismal diathesis.

These forms and types are so distinct that he who fails to take heed of them cannot properly adapt his means to the end in view, and must pursue merely routine methods.

We are greatly aided now in our attempts to arrive at just conclusions respecting the therapeutical value of our remedies for rheumatism by the exact knowledge we possess of the natural history of this disease. Thanks to the "mint-water treatment" of Guy's Hospital, we know that rheumatism has a tendency to get well about the fourteenth day, and again, but more decidedly, about the twenty-first day, but that it usually continues on to the sixth week and does not really cease earlier, as I think Dr. Fuller conclusively shows. The traditional "six weeks and blankets," under the spoliative treatment formerly employed, seems to be about the natural limit of rheumatism, and hence, if under our remedies the duration of the disease is distinctly less, they have exerted a curative influence. It is very apparent, therefore, that we have several remedies which possess curative value in this disease, for under their use the duration of it is materially abbreviated.

Taking up for consideration, first, the type of feeble, anæmic, nervous subject—what method shall we pursue? If I were governed merely by the fashion of the time I would direct salicylic acid or salicin—an undeniably efficient remedy in many cases. But in this class of subjects it does not succeed well; they are much depressed by it, and have a tedious convalescence with a strong tendency to relapses. In these cases I decidedly prefer the tincture of the chloride of iron, in half-drachm doses, well diluted with water. We owe chiefly to Dr. Russel Reynolds the important fact that the tincture of iron is an efficient remedy in acute rheumatism. It cuts short the duration of the disease, and, what is even more important, lessens the danger of cardiac complications. Dr. Anstie pointed out another fact—that the tincture of iron has the power of prophylaxis, of preventing attacks that are impending. Whether it acts by virtue of its acid or its iron is not known, but it is probably the former. Dr. Ridge has shown that the mineral acids are decidedly curative in acute rheumatism. Alkalies are curative in rheumatism! mineral acids are curative in rheumatism! What strange contradiction is this? After all, gentlemen, this opposition of agents is more apparent than real. It is not difficult to conceive, that whilst alkalies neutralize the acid of rheumatism, the mineral acids may prevent its formation. We may, therefore, assume that the virtues of the chloride of iron are due to its acid; but we should not obtain the same good results from chlorhydric acid, for iron aids in the restoration of the blood, and is useful for this reason.

I direct, as I have already indicated, and as you have witnessed, thirty minims of the tincture well diluted with water, every four hours. The affected joints are wrapped in cotton if the patient desires it, but otherwise are simply kept at rest, and if the pain is severe, some small blisters are applied around the joint, but not on it. I have treated many cases with the iron alone, and with iron aided by moderate doses of alkalies and blisters. The best results have been obtained in these weak and anæmic subjects by the iron and blisters, and an occasional laxative of Rochelle salt. The treatment by blisters alone is a highly efficient plan, and is by no means so painful and disagreeable as it appears at first sight. The blisters remarkably relieve the pain, and patients soon learn this and ask for their repetition. But the blisters do more: they bring about a more alkaline condition of the blood, and render the urine less acid, or bring it to neutral, or even to alkaline. I do not, as the French physician (Dr. Dechilly) who proposed the method, apply large blisters over the whole of the affected joints, but as Dr. Davies, of the London Hospital, who introduced the method into England, apply smaller blisters to encompass the joints. To be more explicit: I have small blisters, the size of a silver dollar, placed around the joint, leaving an interval between for succeeding applications.

In these weak subjects a few blisters are applied, and the joint is supported at rest, but the tincture of iron is the chief remedy. Managed in this way, the duration of the cases rarely exceeds two weeks; heart complications are infrequent, and the patient's strength is conserved so that convalescence is rapid and relapses uncommon.

The cases of the second class require different management. They are the fat and flabby subjects, often excessive consumers of malt liquors, who suffer habitually with acid indigestion and the usual concomitants of this state. Such subjects present a delusive appearance of good health, but they have a weak circulation, are easily put out of breath, tire on the least exertion, and often suffer from lumbago, myalgia, and other so-called rheumatic troubles. When attacked with acute rheumatism, they are very apt to have endo- or exo-cardial complications. These cases are most successfully treated by the alkaline plan. In, I believe, almost the last paper written by the late Dr. Fuller, which was in opposition to Drs. Gull and Sutton's "mint-water treatment," he insisted strongly on certain points in regard to the use of alkalies, inattention to which had been the cause of failure in the treatment. He says we must give not less than an ounce and a half of the alkaline carbonates, either alone or in combination with a vegetable acid, during the first twenty-four hours of the treatment. This may be prescribed as a drink—a lemonade—by adding lemon juice or citric acid to the solution of the carbonates—two drachms of the carbonates, an ounce of lemon juice, or half a drachm of citric acid dissolved in four ounces of water, and taken every three or four hours. If the bowels are constipated, he gives compound cathartic pills at bed-time. As soon as the urine, when passed, ceases to exhibit an acid reaction, he reduces the alkali one-half. This reduction of the daily quantity of alkali goes on each day, until the fourth or fifth day, when, if the urine continues to be alkaline, he prescribes bark preparations or quinia, at the same time continuing the alkalies in moderate quantity. If treated on this plan, the class of cases under consideration get well within two weeks, and are often up in a week. Instead of giving the quinia in the small doses of three grains advised by Dr. Fuller, the results are much better if twice that quantity is given every four hours. In these cases, instead of quinia I usually give, after the alkali course, the tincture of iron; and, if the attack is a severe one, apply blisters about the principal joints.

The third group of cases consists of vigorous subjects having, in a considerable portion of them, an inherited tendency. According to my experience, cases of this type are adapted to the action of salicylic acid, and are often relieved with remarkable promptitude by means of it. Salicin is probably nearly as effective, but it must needs be given in such quantity as to be difficult to manage. Scruple doses of salicylic acid seem to be sufficient for most cases of rheumatism, provided they are often enough repeated. The patient should receive not less than two drachms every twenty-four hours, and considerably more may be required. I have found that salicylic acid is more effective if given in solution or contemporaneously with an excess of alkali than if administered in powder by itself. If kept for a few hours in solution with sodium bicarbonate in excess, the solution becomes brownish or greenish-brown, and emits an odor of wintergreen. Take it all in all, the most satisfactory procedure is to give wafers containing the salicylic acid, and alternate with an effervescing draught of an alkaline carbonate—the officinal effervescing powder answers the purpose. The amount of relief given by this remedy in many cases is amazing, and in a few hours, a cure being effected not unfrequently in three or four days. When good is being accomplished by it, the evidence is quickly afforded in relief to pain and decline of temperature. If, therefore, after several days—three or four—persistent and efficient administration of salicylic acid, the signs of improvement are wanting, it is probable that nothing will be accomplished by its continued use. If the stomach will not bear it, or if the considerable doses necessary depress the action of the heart, or cause great irregularity in the pulsations, it must be discontinued.

Notwithstanding the importance of these remedies or methods of treatment, there are accessories scarcely inferior in the influence which they exert over the progress of the case. The diet must be carefully regulated. Solid food of any kind seems to be hurtful, and there is usually great repugnance to it. Milk and beef, mutton or chicken broth, are the chief components of the diet. Large draughts of milk are useful by maintaining free action of the kidneys. Coffee and tea may be allowed, but wine, beer and spirits are highly injurious.

Shall any attention be given to the joints? Experience does not justify the local treatment of the rheumatic inflammation. The curative effects of blisters are not due to the notion at one time entertained, of the withdrawal of a morbific material from the affected parts, or to the counter-irritant action, but to their systemic effects in increasing the alkalinity of the blood, and lessening the acidity of the urine, and their power to relieve pain. Wrapping the joints in cotton is comforting to the patient, but it is questionable practice, as the heat is retained, and the temperature of the joints kept above that of the neighboring parts. The application of alkaline lotions, at one time much used, owing to the theoretical notions then entertained, is now rarely employed. Painting with iodine tincture does not influence the course of the case in any way. To maintain immobility of the affected joints, is a measure of the highest utility. Motion increases the pain and swelling, which react in turn on the systemic state, and conversely, an absolutely quiescent state of the joints diminishes pain and lessens fever. To secure the necessary quietude has been attempted by mechanical means—by starch or plaster bandages; but there are many joints so situated that this method, if desirable, would be impracticable. In fact, the desired immobility can be secured only by moral and medicinal means. The necessity for quiet—for absolute quiet—should be impressed on the patient, but moral suasion must be aided by means to quiet pain and restlessness. It is the sedative influence of the bromides on the centres of conscious impressions, and on the reflex and motor centres, which gives them importance as remedies in acute rheumatism, and by some of our best authorities they are assigned the highest place.

Relief to pain and restlessness is best afforded by the agents which exert a curative influence, but if pain persists relief must be given in some other way—by anodynes. If the bromides are active enough to allay pain, to bring sleep, and to quiet the restlessness, they are to be preferred; but it will generally be found, I think, that they do not possess sufficient anodyne power. Morphia or Dover's powder are usually resorted to, but the relief which they afford is at the expense of a protracted convalescence. By checking elimination, opium retards improvement. There is an agent which happens to have a decided effect in relieving pain, while at the same time it promotes elimination—that is, atropia; which, for this purpose, was first used and recommended by Dr. Harley. It should be administered hypodermically and in the neighborhood of the affected joints. The dose for each injection need rarely exceed the one-eightieth grain a day.

I have probably occupied sufficient time in giving this summary of the treatment of rheumatism, yet I ought to say something of important complications. It is by no means an unusual circumstance to have endo- or exo-cardial inflammations occur—in, probably, one-third of all the cases. To combat it, there are three remedies of chief value—morphia, ammonia, and digitalis. As soon as the fact of the cardiac complication having arisen is known, the carbonate of ammonia in solution of the acetate (five grains to a tablespoonful) should be freely given, with the object of securing prompt solution of the fibrinous exudation of deposited fibrin. To check the inflammatory process, and lessen the work of the heart, morphia and digitalis are prescribed. The morphia is most efficient when administered hypodermically, and the digitalis when in the form of infusion. As there is no therapeutical incompatibility, these agents may be given contemporaneously. When the acute symptoms subside, to relieve the immediate and prevent the ulterior bad effects of the inflammation, the tincture of iron and quinia should be given freely, and the heart should be kept steady by digitalis. The extent to which restoration of these injured parts, delicate in structure as they are, can be carried by rightly seconding the efforts of nature, is very surprising. Shall counter-irritants be used? Although we are told that a blister applied to the bony walls of the chest cannot affect the condition of organs within, yet experience is in favor of the practice, and the patient's subjective sensation of relief is more valuable testimony than the deductions of theory. Neither need we be concerned about the blistering point, but put on one, not over the præcordia to interfere with auscultation, but on the side of the chest, in the subaxillary space.

There is a complication of rheumatism—fortunately very rare—in which, without any apparent cause, the temperature suddenly leaps up to 106°, 108°, even 109° Fahr. This state of *hyperpyrexia*, as it is called, is accompanied by delirium and by cardiac and respiratory disturbances. That the grave symptoms of hyperpyrexia are due to the high temperature, is now admitted on all sides, but no adequate explanation has thus far been given of the causes producing it. We only know that in some cases, hyperpyrexia comes on, and paralysis of brain and heart quickly ensues if the excess of heat cannot be removed. Until the value of the cold bath had been made known, there existed no means of diminishing the extraordinary heat, and these cases were always fatal. Now, however, the cold bath affords us the means of rescuing some cases from impending death. The method of the application is the same as for fevers, but if the bath is not available, the wet-pack is a resource which can always be utilized.

